# Protective role of thymoquinone in hyperlipidemia-induced liver injury in LDL-R^−/−^mice

**DOI:** 10.1186/s12876-023-02895-0

**Published:** 2023-08-11

**Authors:** Fei Wang, Wei Yao, Dexin Yu, Yuhua Hao, Yuling Wu, Xiaoqing Zhang

**Affiliations:** 1 Department of Gastroenterology, Affiliated Zhongshan Hospital of Dalian University, No. 6 Jiefang Street, Dalian, China; 2 Department of Internal Medicine, Affiliated Zhongshan Hospital of Dalian University, No. 6 Jiefang Street, Dalian, China; 3 Department of Injection, Affiliated Zhongshan Hospital of Dalian University, No. 6 Jiefang Street, Dalian, China

**Keywords:** Hyperlipidemia, Thymoquinone, Liver injury, Pyroptosis, LDL-R^−/−^ mice, Western blotting

## Abstract

**Background:**

Hyperlipidemia, a heterogeneous group of disorders characterized by elevated plasma lipids in the blood, causes severe health problems, leading to fatty liver disease and nonalcoholic fatty liver disease. Thymoquinone, the major active chemical component of Nigella sativa, reportedly exerts a vast array of biological effects. Various studies have reported that Thymoquinone protects against liver injury.

**Aims:**

The aim of this study was to investigate the possible protective effects of Thymoquinone against liver injury in hyperlipidemia-induced LDL-R^−/−^ mice.

**Methods:**

Eight-week-old male LDL-R^−/−^ mice were randomly divided into three groups: a control group fed a normal diet and two groups fed a high-cholesterol diet or high-cholesterol diet mixed with Thymoquinone. All groups were fed different diets for 8 weeks. Blood samples were obtained from the inferior vena cava and collected in serum tubes. The samples were then stored at − 80 °C until used. Longitudinal sections of liver tissues were fixed in 10% formalin and then embedded in paraffin for histological evaluation. The remainder of the liver tissues were snap-frozen in liquid nitrogen for reverse transcription-polymerase chain reaction or western blotting.

**Results:**

Our results demonstrated that Thymoquinone administration significantly reduced liver histological alterations by hyperlipidemia. Thymoquinone mitigated hyperlipidemia-induced liver injury as indicated by the suppression of metabolic characteristics, liver biochemical parameters, pyroptosis indicators, a macrophage marker, and the phosphatidylinositide 3-kinase signaling pathway.

**Conclusions:**

Thymoquinone is a potential therapeutic agent for hyperlipidemia-induced liver injury.

**Supplementary Information:**

The online version contains supplementary material available at 10.1186/s12876-023-02895-0.

## Introduction

Modern living environments and excessive energy intake have led to a tremendous increase in various metabolic diseases such as hyperlipidemia, diabetes, hypertension, and cardiovascular diseases [1]. The consumption of a high-fat diet usually leads to hyperlipidemia, a heterogeneous group of disorders characterized by elevated plasma lipid levels in the blood [2], which cause serious health problems, including atherosclerosis, coronary artery disease, cerebrovascular disease, acute pancreatitis, and nonalcoholic fatty liver disease [3–5]. Hyperlipidemia and its complications are responsible for half of all deaths worldwide. Particularly in China, the situation is extremely serious, with 160 million patients suffering from dyslipidemia [6].

The liver plays a vital role in maintaining systemic lipid homeostasis [7]. Long-term hyperlipidemia may induce liver diseases, including liver steatosis and liver injury. This liver fat accumulation is caused by dysregulated lipid metabolism, including lipid synthesis, fatty acid oxidation, and lipoprotein uptake and secretion in the liver [8]. Recently, several studies revealed that pyroptosis is linked to a high-fat diet [9, 10]. Pyroptosis, a specific programmed cell death, is regarded as an inflammasome-activated process [11]. Nucleotide-binding oligomerization domain-like receptor 3 (NLRP3), the best-studied canonical inflammasome [12], plays a vital role in liver diseases, including ischemia/reperfusion injury, drug-induced hepatotoxicity, and fibrosis [13–15]. The NLRP3 inflammasome is a molecular platform activated by signs of cellular danger that promotes the maturation and secretion of proinflammatory cytokines such as interleukin (IL)-1β and IL-18 [16].

Currently, the aim of major hyperlipidemia treatments is to increase the levels of anti-atherogenic lipoproteins such as high-density lipoprotein (HDL) or decrease those of low-density lipoprotein (LDL), total cholesterol (TC), and triglycerides (TGs) [17, 18]. Lipid-lowering drugs, such as 3-hydroxy-3-methylglutaryl coenzyme A reductase inhibitors (statins), can effectively lower the levels of TC and LDL-cholesterol (LDL-c). However, the adverse reactions of statins, including myopathy and rhabdomyolysis, should be carefully considered [19]. Moreover, a number of clinical cases have reported the hepatotoxicity of statins [20]. Thus, better therapy methods are currently being investigated.

**Fig. 1:**
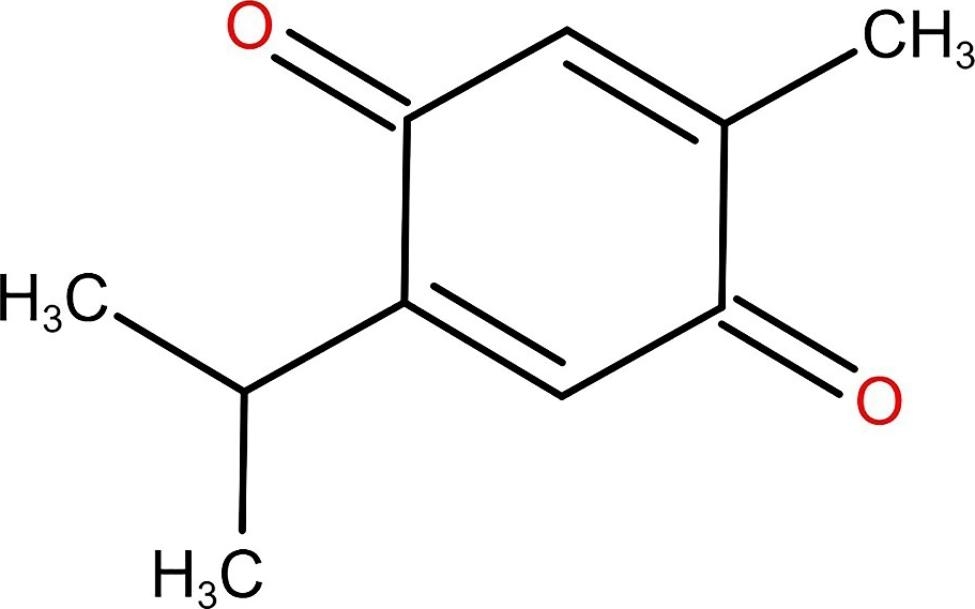
Thymoquinone (2-isopropyl-5-methylbenzo-1, 4-quinone)

Thymoquinone (2-isopropyl-5-methylbenzo-1, 4-quinone) (TQ), the major active chemical component of Nigella sativa, reportedly possesses a vast array of biological effects [21]. The molecular formula of TQ is C10H12O2, and its structure is shown in Fig. 1. A growing number of pharmacological activities of TQ have been investigated, including antioxidant, anti-tumor, antidiabetic, and anti-inflammatory properties, as well as lipid-lowering effects [21]. More recently, the protective effects of TQ on liver injures have been demonstrated experimentally [22]. In addition, a large amount of data shows that TQ has very few adverse effects and a low degree of toxicity [21].

Although a great number of studies about the health-promoting properties of TQ have been published, few studies have evaluated the effects of TQ on hyperlipidemia-induced liver injury. Thus, the aim of the present study was to explore the effects of TQ on hyperlipidemia-induced liver injury in LDL-R^−/−^ mice.

## Materials and methods

### Animal experiments

This study was approved by the Ethical Committee of the Affiliated Zhongshan Hospital of the Dalian University of China. LDL-R^−/−^ mice were purchased from Beijing Vital River Lab Animal Technology Co., Ltd. (Beijing, China). All mice were housed in a room with 12/12-h light-dark cycles at a controlled temperature (24–26 °C). Male LDL-R^−/−^ mice (8 weeks old) were randomly divided into three groups, as follows: mice fed a normal diet (ND group, n = 8), mice fed a high-cholesterol diet (HD group, n = 8), and mice fed a high-cholesterol diet + TQ by gavage (100 mg/kg/d; Sigma-Aldrich, St. Louis, MO, USA) (HD + TQ group, n = 8). The high-cholesterol diet contained 1.5% cholesterol and 15% fat. The experimental diet was purchased from Shanghai Slac Laboratory Animal Co., Ltd. (Shanghai, China). Mice in all groups were fed the appropriate diet for 8 weeks. After 8 weeks, the mice (weight: 25.4–31.3 g) were euthanized with a high dose of pentobarbital (100 mg/kg, intraperitoneally), and lack of respiration and heartbeat was used as an indicator of mouse death [23]. Blood samples were obtained from the inferior vena cava, collected in serum tubes, and stored at − 80 °C until use. The serum was prepared by centrifugation at 3000 rpm for 15 min. Longitudinal sections of the livers were fixed in 10% formalin and embedded in paraffin for histological evaluation. The remaining liver was snap-frozen in liquid nitrogen for mRNA isolation and western blotting analyses. All animal experiments were performed in accordance with ARRIVE guidelines.

### Serum lipoprotein profile

Assay kits (Nanjing Jiancheng Bioengineering Institute, Nanjing, China) were used for detecting TC, LDL-c, TG, alanine aminotransferase (ALT), aspartate aminotransferase (AST), and alkaline phosphatase (ALP), following the manufacturer’s instructions.

### Hematoxylin and eosin staining

Liver tissues (LTs) were fixed by perfusion with 10% buffered formalin. Half of a mice’s LTs were fixed overnight at room temperature, transferred to 70% ethanol, and embedded in paraffin. Paraffin-embedded LTs slices were deparaffinized by immersing them in xylene (thrice, 5 min each), rehydrated in a descending alcohol series (100%, 90%, 80%, and 70% alcohol, 5 min each), dehydrated in an ascending series of ethanol (70%, 80%, 90%, and 100% alcohol, 5 min each), and deparaffinized via immersion in xylene (thrice, 5 min each). Histological changes were detected by staining 5-µm-thick LTs sections with hematoxylin and eosin (H&E) stain according to the manufacturer’s instructions (NO. g1120; Solarbio, Beijing, China). Images were acquired using a B × 40 upright light microscope (Olympus, Tokyo, Japan).

### Immunohistochemistry analysis

Paraffin-embedded LTs were cut into 5 μm-thick cross-sections and deparaffinized prior to staining using a standard protocol. For immunohistochemical staining, LTs were deparaffinized and rehydrated. Next, the sections were blocked with 3% H_2_O_2_ in methanol for 15 min to inactivate endogenous peroxidases and then incubated overnight at 4℃ with one of the following primary antibodies: CD68 (No. 28058-1-AP; Proteintech, Wuhan, China). The sections were then incubated for 30 min at room temperature with a goat anti-rabbit HRP secondary antibody (No. PK10006; Proteintech). All sections were examined under an Olympus B × 40 upright light microscope (Olympus, Tokyo, Japan).

### RNA isolation and real-time RT-qPCR

Total RNA was isolated from LT and complementary DNA (cDNA) was synthesized using the TransScript One-Step gDNA Removal and cDNA Synthesis SuperMix kits (No. AT311-02 Transgen, Beijing, China), respectively, according to the manufacturer’s protocol. Gene expression was quantitatively analyzed by qPCR using TransStart Top Green qPCR SuperMix kit (No. AQ131-01 Transgen, Beijing, China). β-Actin cDNA was amplified and quantitated in each cDNA preparation to normalize the relative amounts of the target genes. Primer sequences are listed in Table 1.

**Table 1:** Primer oligonucleotide sequences

Gene	Primers
IL-1β	F: 5′-TGCCACCTTTTGACAGTGAT-3′
	R: 5′-TGTGCTGCTGCGAGATTTGA-3′
IL-18	F: 5′-ATGGCTGCTGAACCAGTAGAAG-3′
	R: 5′- CAGCCATACCTCTAGGCTGGC-3′
NLRP3	F:5′- CTGCGGACTGTCCCATCAAT-3′
	R:5′- AGGTTGCAGAGCAGGTGCTT-3′
β-actin	F:5′-CGATGCCCTGAGGGTCTTT-3′
	R:5′-TGGATGCCACAGGATTCCAT-3′

Abbreviations: IL-1β: interleukin- 1β; IL-6: interleukin- 6; NLRP3: Nucleotide-binding oligomerization domain-like receptor 3; F: Forward primer; R: Reversed primer

### Western blotting (WB)

Proteins were extracted from LTs using radioimmunoprecipitation assay buffer (P0013B; Beyotime, Shanghai, China). Samples were electrophoresed on 10% SDS-PAGE gel, and proteins were transferred to polyvinylidene fluoride membrane (Immobilon, Millipore, Billerica, MA, USA). Membranes were blocked in Tris-buffered saline with 0.1% Tween-20 containing 5% skim milk and then were incubated in primary antibody diluent (P0023A; Beyotime) and gently shaken overnight at 4 °C. Primary antibodies against NLRP3 (No. A00034-2; Boster, Wuhan, China), IL-18 (No.10663-1-AP; Proteintech), IL-1β (No. ARG56644; Arigo, Hamburg, Germany), PI3K (No.20584-1-AP; Proteintech), and anti-β-actin (No.81115-1-RR; Proteintech). Membranes were then incubated with a secondary antibody (No. 58,802; Cell Signaling Technology) for 1 h at 37 °C. This analysis was carried out independently three times. Protein levels are expressed as protein/β-actin ratios to minimize loading differences. The relative signal intensity was quantified using NIH ImageJ software.

### Statistical analysis

All data are presented as the mean ± SEM. Statistical analysis was performed using SPSS software version 23.0 (SPSS Inc., Chicago, IL, USA). Inter-group variation was measured by one-way ANOVA and subsequent Tukey’s test. The minimal level for significance was *P* < 0.05.

## Results and discussion

## Results

### Metabolic characterization

**Fig. 2:**
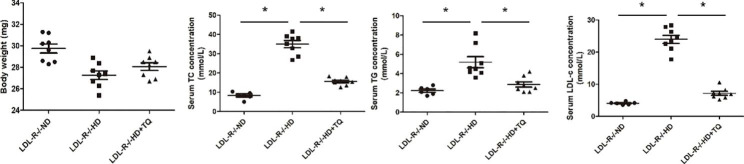
Metabolic data from the three groups after 8 weeks of different treatment. Data are represented as mean ± SEM; n = 8 per group. *P < 0.05 vs. LDL-R^−/−^ HD group. Abbreviations: BW, body weight; TC, total cholesterol; TG, triglycerides; LDL-c, low-density lipoprotein cholesterol

The metabolic characteristics of the three groups of LDL-R^−/−^ mice exposed to treatment are summarized in Fig. 2. The body weight did not vary among the three groups. The HD group exhibited a significant increase in the TC, TG, and LDL-c levels; however, these levels were significantly decreased in the HD + TQ group. There was no variation with respect to the aforementioned parameters in the ND group.

### TQ reduced serum ALT, AST, and ALP levels in the HD group

**Fig. 3:**
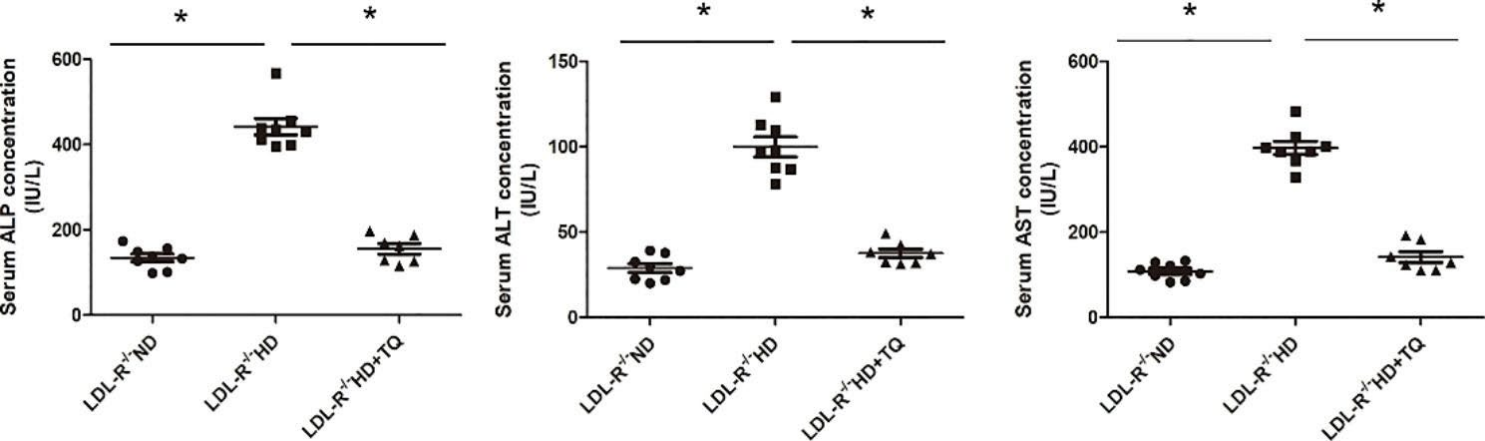
Liver biochemical parameters of the three groups of LDL-R^−/−^ mice fed the different diets. ALT, AST, and ALP levels are presented. Data are represented as mean *±* SEM; n = 8 per group. *P < 0.05 vs. LDL-R^−/−^ HD group

The liver biochemical parameters of the three groups of LDL-R^−/−^ mice are summarized in Fig. 3. The HD group exhibited a marked increase in the ALT, AST, and ALP levels; however, these levels were significantly decreased in the HD + TQ group. There was no variation with respect to the aforementioned parameters in the ND group.

### TQ changed histopathological and immunohistochemical staining in the LTs of the HD group

**Fig. 4:**
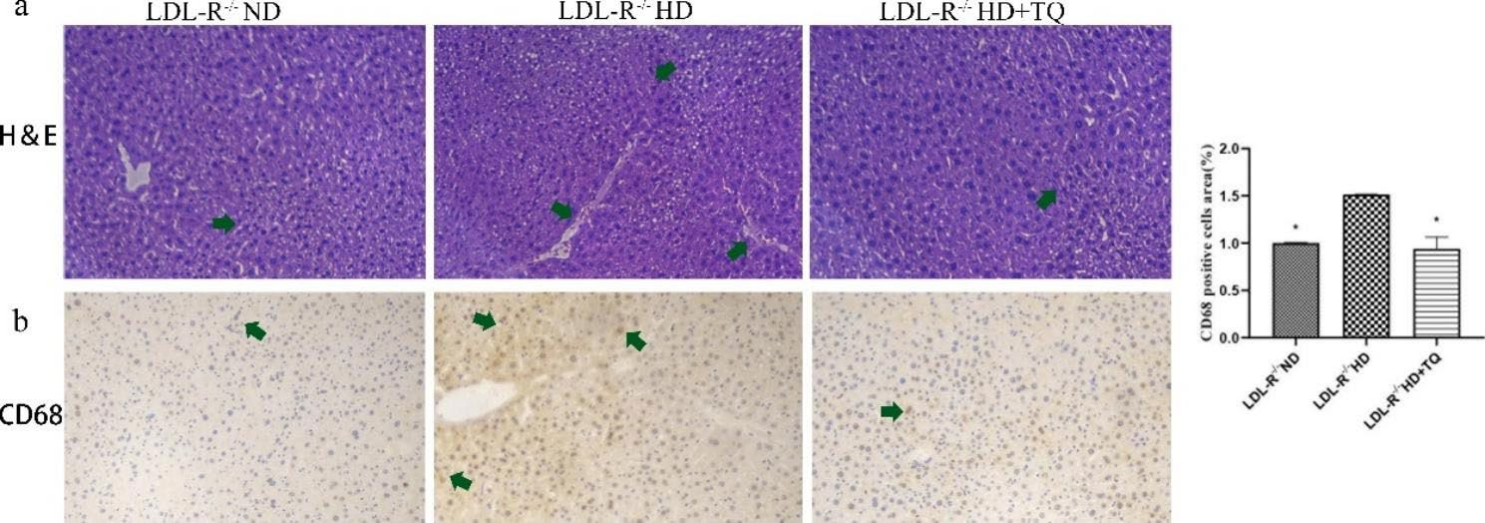
Histopathological changes in the LTs among the three groups of LDL-R^−/−^ mice fed the different diets. **(a-b)** H&E and immunohistochemistry of CD68 in the LTs among the three groups of LDL-R^−/−^ mice fed the different diets. The bar shows the quantification of positive expression. n = 3 per group. Data are presented as the means ± SEM; * *P* < 0.05 vs. the LDL-R^−/−^ HD group

We performed H&E staining to evaluate the histopathological changes in the LT (Fig. 4), and the liver in the ND groups demonstrated the histological sections of normal liver tissues. In contrast, the HD group revealed marked liver injury owing to liver lobule disorder, focal necrosis, swelling of liver cells, and widespread distribution of lipid droplets compared with the ND group. However, the administration of TQ to mice prevented the degenerative changes in the liver structure induced by HD. Immunohistochemical staining with anti-CD68 antibody was performed to evaluate the CD68-positive cells in the LTs (Fig. 4). The HD + TQ group exhibited a markedly reduced accumulation of CD68-positive cells in the LT compared with the HD group.

### TQ reduced pyroptosis-related gene expression in the LTs of the HD group

**Fig. 5:**
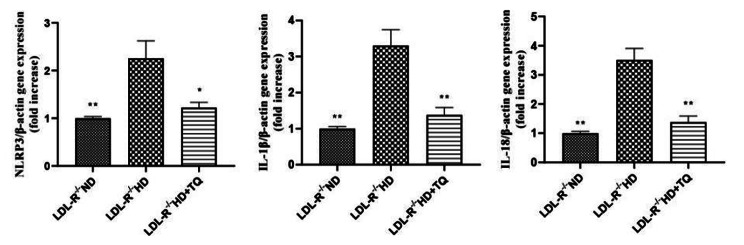
Gene expression of pyroptosis in the LTs obtained from the three groups of LDL-R^−/−^ mice fed the different diets. Relative mRNA expression of NLRP3, IL-1β, and IL-18 in the LTs. Data are represented as mean ± SEM; n = 3 in each group. *P < 0.05, **P < 0.01 vs. LDL-R^−/−^ HD group

To examine the involvement of pyroptosis-related gene expression in the LTs obtained from mice that belong to the three experimental groups, the expression of NLRP3, IL-1β, and IL-18 was quantified using qPCR (Fig. 5). Compared with ND group, NLRP3, IL-1β, and IL-18 expression was upregulated in the HD group; however, this upregulation was attenuated in the HD + TQ group.

### TQ decreased pyroptosis-related protein expression in the LTs

**Fig. 6:**
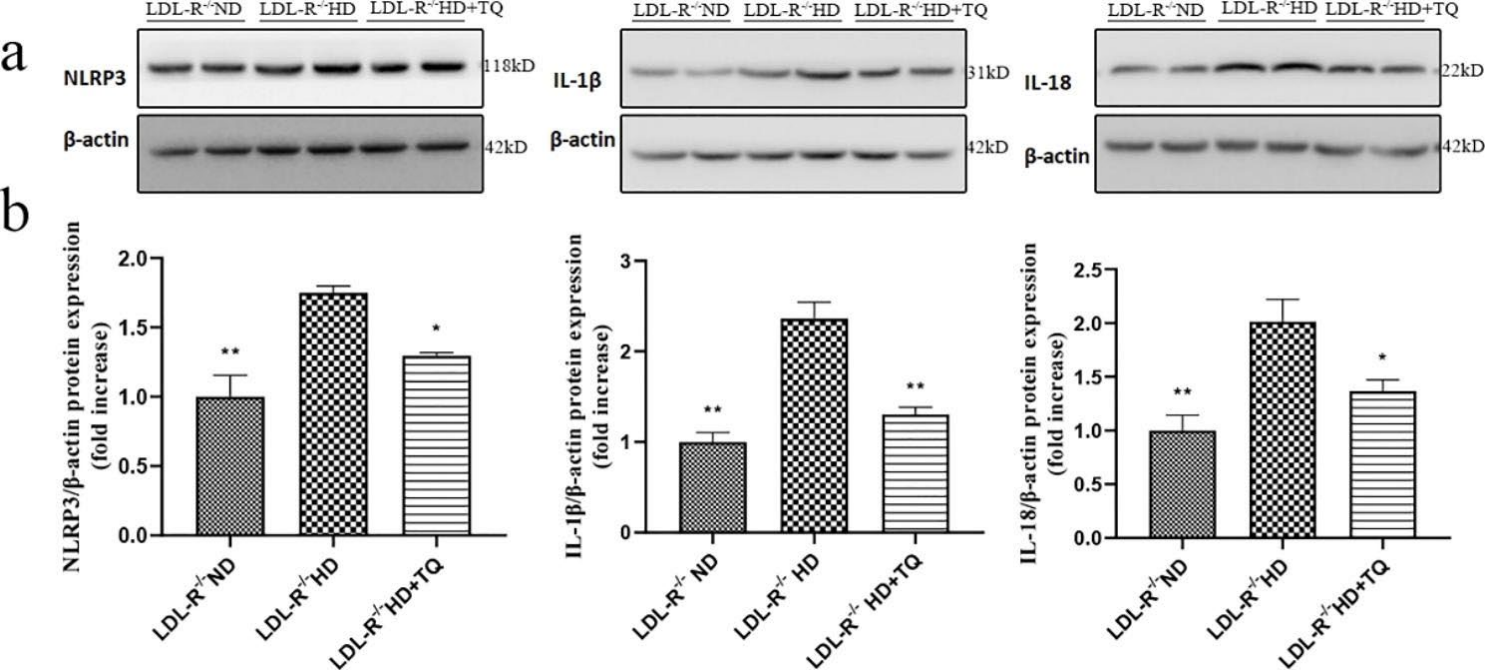
NLRP3, IL-1β, and IL-18 expression levels in the LT among the three groups of LDL-R^−/−^ mice fed the different diets. **(a)** WB to detect the NLRP3, IL-1β, and IL-18 expression levels in the LT. The blots edge was imprinted as a marker and were cut prior to hybridization with antibodies. **(b)** Bar graph depicts the quantification of NLRP3, IL-1β, and IL-18 expression levels. Data are expressed as mean ± SEM; n = 3 in each group. *P < 0.05, **P < 0.01 vs. LDL-R^−/−^ HD group

We performed WB to quantify NLRP3, IL-1β, and IL-18 expression levels in the LTs (Fig. 6). The HD + TQ group exhibited a marked decrease in NLRP3, IL-1β, and IL-18 expression levels in the LT compared with that observed in the HD group. These results indicate that TQ decreases NLRP3, IL-1β, and IL-18 expression levels in the HD + TQ group.

### TQ reduced phosphatidylinositide 3-kinase (PI3K) expression in the damaged LTs

**Fig. 7:**
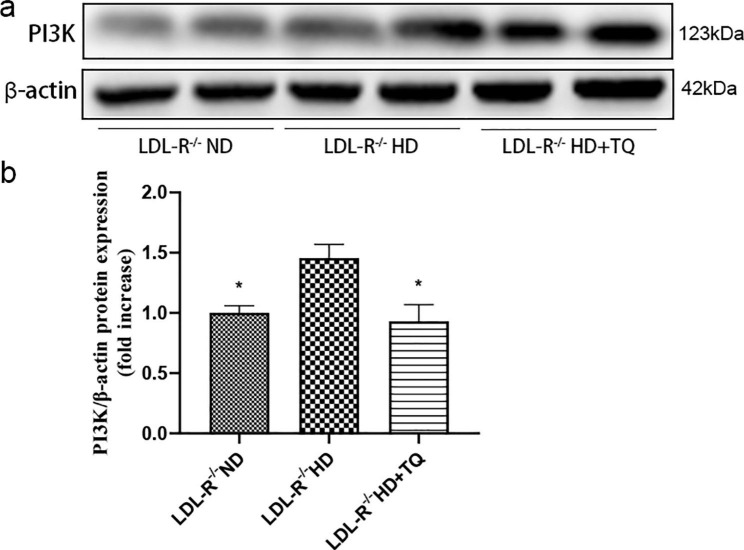
PI3K levels in the LTs of three groups of LDL-R^−/−^ mice fed the different diets. WB to detect the PI3K level in the LTs. **(a)** The blots edge was imprinted as a marker and were cut prior to hybridization with antibodies. **(b)** Bar graph depicts the quantification of PI3K expression level. Data are represented as mean ± SEM; n = 3 in each group. *P < 0.05 vs. LDL-R^−/−^ HD group

To investigate the effect of TQ on the regulation of the PI3K signaling pathway, we analyzed the PI3K levels in the three mice groups by performing WB (Fig. 7). We found a higher PI3K expression level in the HD group than in the ND group. Additionally, the HD + TQ group exhibited significantly lower PI3K levels than the HD group (Fig. 7).

**Fig. 8:**
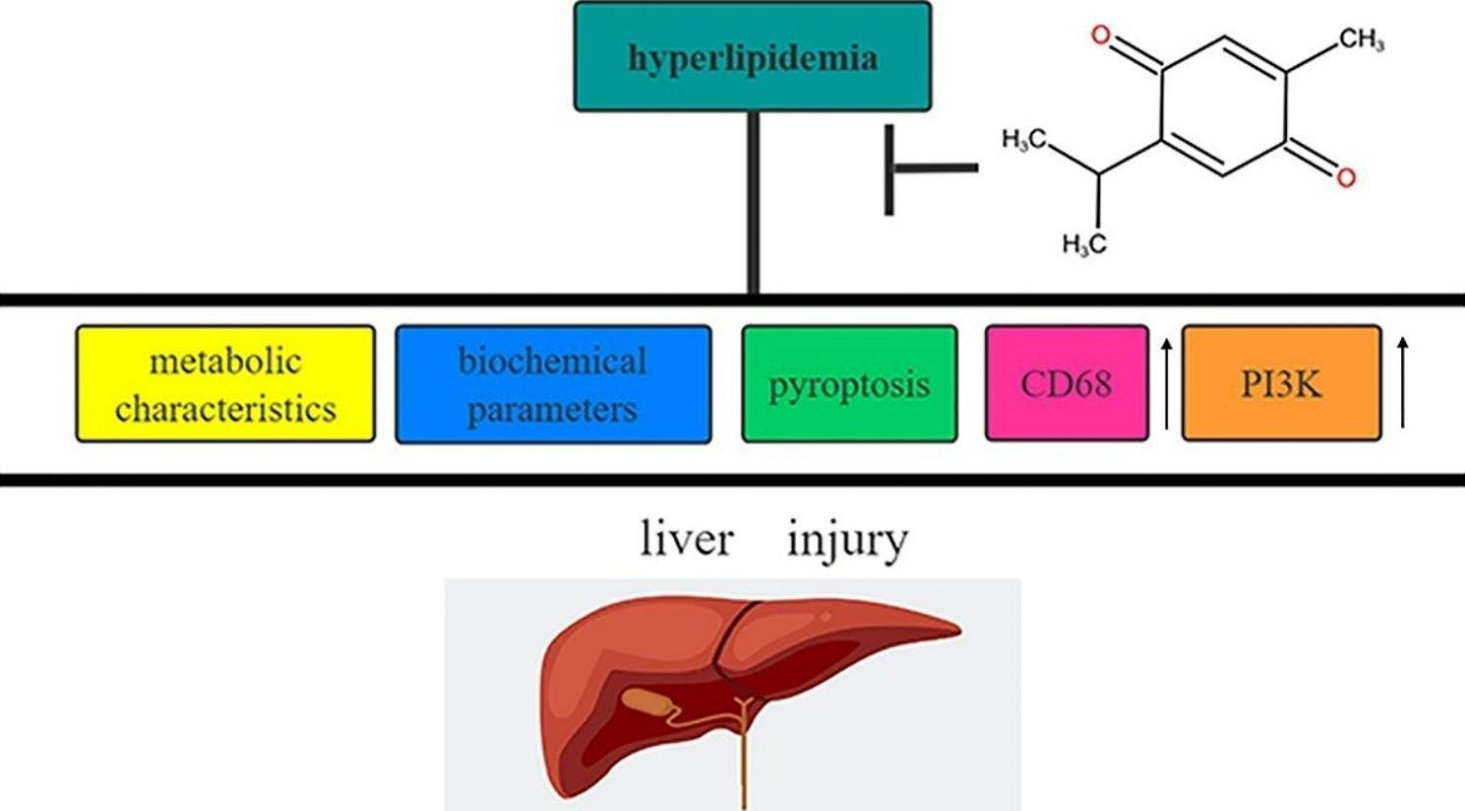
Schematic diagram showing how TQ protects against liver injury by altering metabolic characteristics, liver biochemical parameters, pyroptosis indicators CD68 and PI3K expression elicited by hyperlipidemia

## Discussion

This study illustrated that TQ has a protective effect against liver injury by altering metabolic characteristics, liver biochemical parameters, pyroptosis indicators, macrophage accumulation and secretion, and signaling pathways elicited by hyperlipidemia. These findings are summarized in Fig. 8. Among metabolic characteristics, we found that TC, TG, and LDL-c levels were higher in the HD group than in the ND group of LDL-R^−/−^ mice. Interestingly, TC, TG, and LDL-c levels were significantly lower in the HD + TQ group than in the HD group, which agrees with what Ragheb et al. [24] reported in high-cholesterol diet rabbit models.

The hyperlipidemia-induced liver injury could further be investigated by the detection of liver biochemical parameters (AST, ALT, and ALP). This study indicated that the serum AST, ALT, and ALP levels were significantly higher in the HD group than in the ND group. Li et al. [25] reported similar results in a high-fructose-fed Kunming mouse model. Furthermore, it was interesting that the HD + TQ group exhibited a prominent reduction in these liver biochemical parameters compared with the HD group. Accordingly, several studies have revealed that TQ treatment leads to similar results regarding the serum AST, ALT, and ALP levels in diazinon-induced liver toxicity in vivo and liver injury induced by anti-tuberculosis drugs in murine models [26].

Moreover, according to the histological evidence of liver injury as assessed by performing H&E staining, treatment of murine models with TQ completely protected mice against the liver lobule disorder, focal necrosis, swelling of liver cells, and widespread distribution of lipid droplets caused by hyperlipidemia. Hyperlipidemia-induced liver injury is usually associated with an increase in the number of macrophages. Macrophage-derived foam cells release cytokines that recruit more macrophages to lesions and influence lipid deposition [27]. The marker CD68 identifies macrophages. CD68-positive cells are found in hyperlipidemia-damaged LT [28]. In the present study, we performed immunohistochemical staining to evaluate the number of macrophages in the LT and showed that the number of CD68-positive cells was significantly higher in the HD group than in the ND group of LDL-R^−/−^ mice. However, mice in the HD + TQ group showed markedly less accumulation of CD68-positive cells in the LT than those in the HD group. This elucidated that TQ reduced macrophage accumulation in the LT of the HD group mice.

Recently, several studies revealed that pyroptosis is linked to a high-fat diet. Pyroptosis is a specific programmed cell death characterized by inflammatory cytokine release. NLRP3 is the best-studied canonical inflammasome. The role of NLRP3 inflammasome activation has been paid widespread attention in liver diseases, including ischemia/reperfusion injury, drug-induced hepatotoxicity, and fibrosis. NLRP3 inflammasome activation results in the secretion of inflammatory cytokines, such as IL-1β and IL-18. Chen and colleagues [29] established the formation and activation of the NLRP3 inflammasome and IL-1β in nonalcoholic steatohepatitis. Additionally, Hendrikx et al. [30] reported that IL-18 and IL-1β gene expression is increased in hyperlipidemic mice. A previous study [31] demonstrated that TQ significantly inhibits NLRP3, IL-1β, and IL-18 expression in CLP-induced septic cardiac damage. Moreover, Suguna et al. [32] revealed that TQ suppresses the gene expression level of IL-1β, IL-18, and NLRP3 in HFD-fed rats. Here, we showed markedly reduced NLRP3, IL-1β, and IL-18 expression in the HD + TQ group, as evidenced by RT-PCR and western blottin in the LTs, compared with the HD group. These results indicate that TQ also downregulated pyroptosis in the HD group.

Furthermore, to investigate the effect of TQ on the regulation of the PI3K signaling pathway, we performed western blotting to assess the PI3K level. PI3K represents a key signaling molecule that regulates several cellular functions, including proliferation, survival, adhesion, and migration, in the liver [33, 34]. Hu and colleagues showed significantly increased PI3K expression in the murine liver following aluminum overload [35]. A recent study indicated that TQ markedly increases cisplatin-induced anti-tumor effects on gastric cancer through PI3K/AKT signaling pathway inhibition [36]. In addition, Bai and colleagues established that TQ significantly inhibits PI3K expressing in thioacetamide- and lipopolysaccharide-induced liver fibrosis and inflammation [37, 38]. In the course of other diseases, Wang et al. [39] demonstrated that TQ inhibits lipopolysaccharide-induced PI3K in BV2 microglial cells. In our study, we observed a higher PI3K expression level in the HD group than in the ND group. Conversely, the HD + TQ group showed a reduced PI3K level compared with the HD group. Taken together, TQ might inhibit PI3K signaling to protect against hyperlipidemia-induced liver injury.

## Conclusions

In conclusion, our study elucidated that TQ mitigated hyperlipidemia-induced liver injury as indicated by the suppression of metabolic characteristics (TC, TG, and LDL-c), liver biochemical parameters (ALT, AST, and ALP), pyroptosis indicators (NLRP3, IL-1β, and IL-18), a macrophage marker (CD68), and the PI3K signaling pathway. These findings provide novel insights into the role of TQ in hyperlipidemia-induced liver injury and raise the possibility of developing new therapeutic interventions to treat liver injury.

### Limitations

However, some limitations need to be noted. First, mice were randomly divided into 3 groups, we did not set a separate TQ group, therefore, the effect of TQ in ND group mice didn’t know. Second, we are not performed cell experiments to explore the protection mechanism of specific pathways of TQ on fatty liver injury. Further studies are needed to confirm this.

### Electronic supplementary material

Below is the link to the electronic supplementary material.

Supplementary Material 1

## Data Availability

The datasets used and/or analyzed during the present study are available from the corresponding author on reasonable request.
