# Supplementary figures and images for: Protective role of thymoquinone in hyperlipidemia-induced liver injury in LDL-R−/−mice
Source: BMC Gastroenterol. 2023 Aug 11;23:276. doi: 10.1186/s12876-023-02895-0 (PMC10416449; doi:10.1186/s12876-023-02895-0)

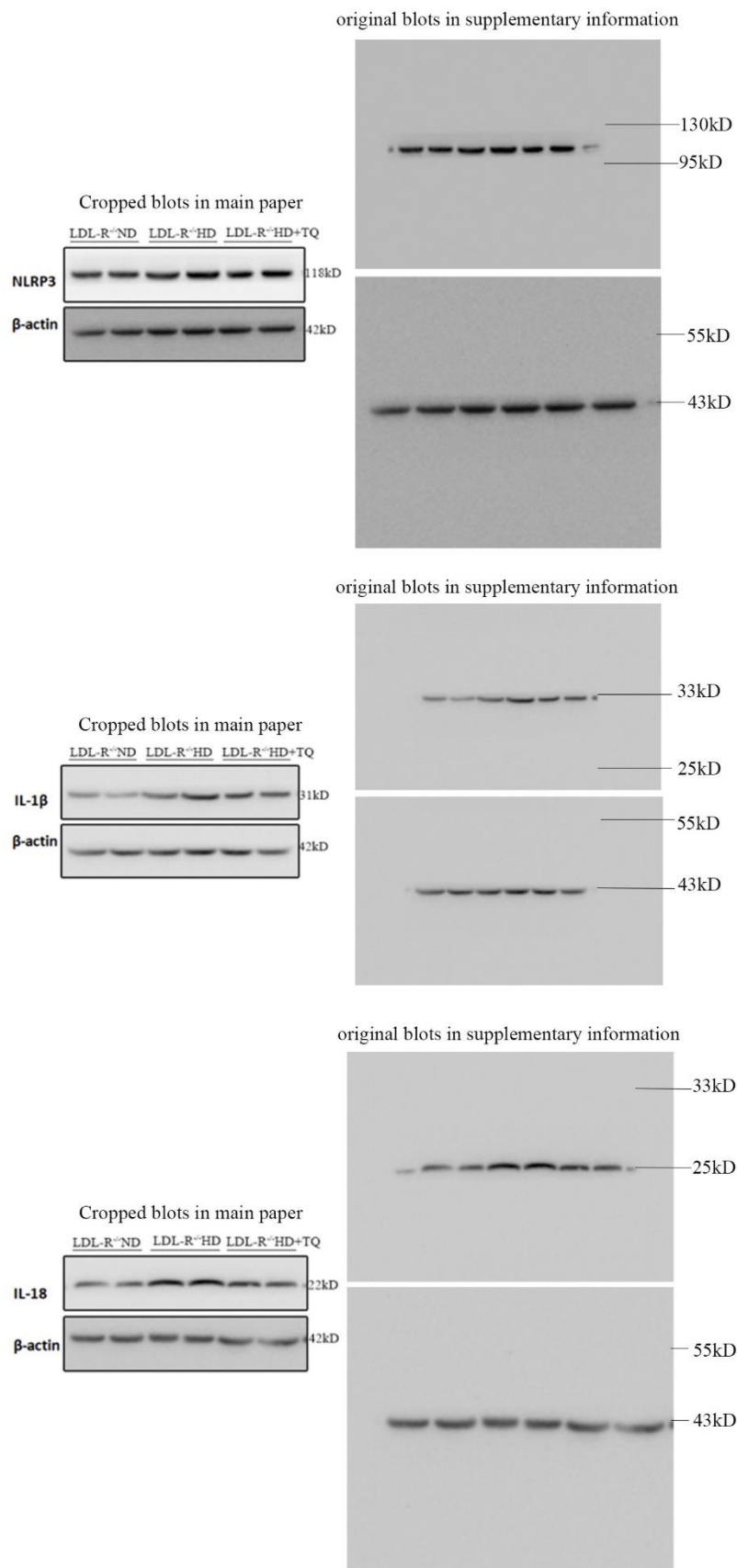

Figure S6. The NLRP3, IL-1 $\beta$ , and IL-18 original gels.

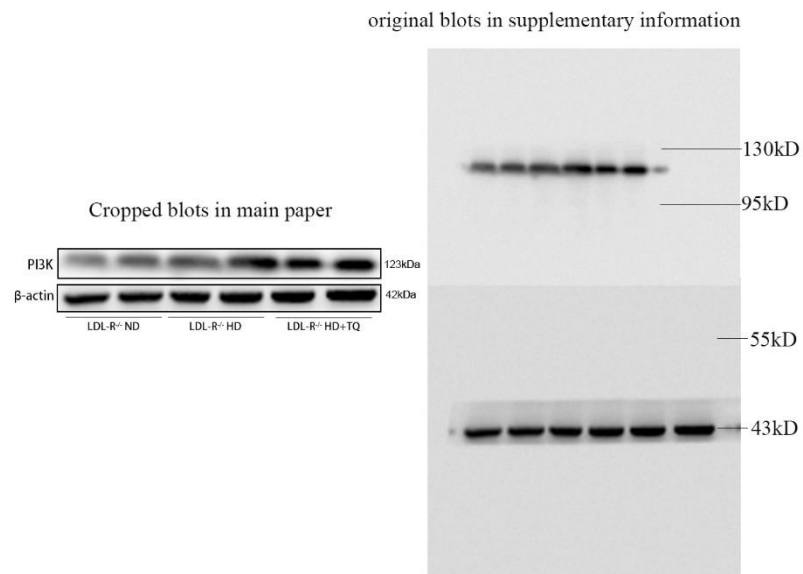

Figure S7. The PI3K original gels.

Supplement: Supplementary file 1 — Supplementary Material 1 [file 12876_2023_2895_MOESM1_ESM.pdf]
